# Why Do Children From Age 4 Fail True Belief Tasks? A Decision Experiment Testing Competence Versus Performance Limitation Accounts

**DOI:** 10.1111/cogs.70069

**Published:** 2025-06-06

**Authors:** Lydia Paulin Schidelko, Hannes Rakoczy

**Affiliations:** ^1^ Department of Developmental Psychology University of Göttingen

**Keywords:** Theory of Mind, Meta‐representation, True belief task, False belief task, Pragmatics

## Abstract

The standard view on Theory of Mind (ToM) is that the mastery of the false belief (FB) task around age 4 marks the ontogenetic emergence of full‐fledged meta‐representational ToM. Recently, a puzzling finding has emerged: Once children master the FB task, they begin to fail true belief (TB) control tasks. This finding threatens the validity of FB tasks and the standard view.  Here, we test two prominent attempts to explain the puzzling findings against each other. The perceptual access reasoning account (a competence limitation account) assumes that children at age 4 do not yet engage in meta‐representation, but use simpler heuristics (“if an agent has perceptual access, she knows and then acts successfully; otherwise, she acts unsuccessfully”). In contrast, the pragmatics approach (a performance limitation account) suggests that children at age 4 do have meta‐representational ToM but are confused by pragmatic task factors of the TB task. The current study tested competing predictions of both accounts in a decision experiment. Results from 165 4‐ to 7‐year‐olds reveal that failure in the TB task disappeared once the tasks were modified: children mastered both FB and TB tasks when the latter were adapted in terms of heuristic and pragmatic factors. Importantly, this pattern held in conditions in which the pragmatics account predicts success, but the perceptual access account predicts failure. Overall, the present findings thus corroborate the standard view (children use meta‐representational ToM from age 4, at the latest) and suggest that difficulties with TB tasks merely reflect pragmatic performance factors.

## 1 Introduction

Theory of Mind (ToM) is the ability to ascribe mental states, such as beliefs and desires, to oneself and to others, in the service of action explanation and prediction (Premack & Woodruff, 1978). At the conceptual heart of ToM lies meta‐representation: representing how agents represent the world and act accordingly. The litmus test of meta‐representation in developmental research is the false belief (FB) task which requires children to ascribe a mistaken belief to another agent and predict her actions accordingly. In its classic version, the change‐of‐location FB task, a story protagonist sees an object being put into one location before the object is moved to another location in the protagonist's absence. Children are then asked to predict where the protagonist will look for the object upon her return (Wimmer & Perner, 1983).

Numerous studies have consistently demonstrated that children begin to solve this task around the age of 4 (Wellman, Cross, & Watson, 2001). Children younger than 4 tend to systematically fail FB tasks by predicting that the protagonist will look for the object where it actually is. Success in the FB task goes along with emerging competence in conceptually related tasks that all require an understanding of (mis‐)representations (e.g., Bowler, Briskman, Gurvidi, & Fornells‐Ambrojo, 2005; Iao & Leekam, 2014; Leekam, Perner, Healey, & Sewell, 2008; Sabbagh, Moses, & Shiverick, 2006, Exp. 2; Sabbagh, Xu, Carlson, Moses, & Lee, 2006). This conceptual transition at age 4 is widely interpreted as the emergence of a novel capacity for meta‐representational thinking (Perner, 1991; Perner & Roessler, 2012; Rakoczy, 2022).

### 1.1 Puzzling performance pattern in true belief control tasks

However, this standard picture of the emergence of meta‐representational ToM around age 4 has recently come under pressure.^1^ Puzzling findings from true belief (TB) control tasks raise the serious possibility that children's success in FB tasks reflects false positives: children may not solve these tasks on the basis of true meta‐representation, but merely by using simpler heuristics. The relevant findings are the following: the TB condition is structurally like FB tasks with the only difference that the protagonist watches not only the object's initial placement but also its relocation and thus holds a TB about the object's final location. TB tasks were originally devised for younger children who fail the FB task in order to control for the possibility that this failure results from overall difficulties with understanding the narrative structure of the task. In this vein, many studies have shown that children below age 4 who fail the FB condition do answer the TB condition correctly. Recent evidence from children of a broader age range, however, shows puzzling patterns of performance: children from age 4 on who come to solve FB tasks suddenly begin to fail the TB task. Children now consistently predict that the protagonist—despite the fact that she observed the location change—will look for her object in the first location. Only from around age 8−10, they recover from the failure, come to solve the TB task again, and thus consistently master both FB and TB tasks (Fabricius, Boyer, Weimer, & Carroll, 2010, 2021; Friedman, Griffin, Brownell, & Winner, 2003; Hedger & Fabricius, 2011; Huemer et al., 2023; Oktay‐Gür & Rakoczy, 2017; Rakoczy & Oktay‐Gür, 2020; Schidelko, Proft, & Rakoczy, 2022b). A substantial number of studies from different labs thus converge in showing a U‐shaped curve in TB performance marked by three phases: Children younger than 4 tend to pass TB and fail FB tasks; children from around 4 to around 8−10 years show the reverse pattern; and only from around age 8−10 do children then consistently pass both FB and TB tasks.

Different theoretical approaches, however, diverge in the way they attempt to explain this U‐curve. In particular, they diverge with respect to the implications of these unexpected U‐curve patterns in TB tasks for the standard picture of the emergence of meta‐representational ToM. On one view, this pattern indicates a deep conceptual competence limitation and thus in fact refutes the standard picture: before they consistently master both TB and FB tasks, children do not really operate with meta‐representational concepts of beliefs and other propositional attitudes, but merely with simpler heuristics (Fabricius et al., 2021). In contrast to such conceptual competence limitation accounts, the opposing view interprets the puzzling TB findings merely as an indicator of pragmatic performance limitations: The standard picture is correct; young children from age 4 do operate with meta‐representational capacities (Rakoczy & Oktay‐Gür, 2020; Schidelko et al., 2022b). But these capacities are masked in some tasks by excessive pragmatic or other performance factors. We portray both positions in detail in the following, describe how they both are compatible with most existing data, and then discuss how we can go beyond this and test them against each other.

#### 1.1.1 Competence limitation accounts: TB failure reflects lack of ToM

According to conceptual competence limitation accounts, the puzzling TB findings suggest that children age 4−6 or even older do not yet have proper meta‐representational competence; rather, they merely use simpler heuristics that may look like true meta‐representation but are not. Such heuristics may mimic proper belief ascription and produce correct answers in FB tasks—but for the wrong reasons. At the same time, these heuristics produce incorrect answers in TB tasks. One such account assumes that children undergo three phases, the first two characterized by simple heuristics, and only the third characterized by true ToM (Fabricius et al., 2010; Hedger & Fabricius, 2011).

- Phase 1: Before age 4, children use reality‐based reasoning strategies, assuming agents will search for objects where they really are, and so on.
- Phase 2: From age 4 on, until around age 6, children then also have a so‐called “perceptual access heuristic” at their disposal which is characterized by two simple rules. First, agents with perceptual access possess knowledge and agents who lack perceptual access lack knowledge (“does see → does know” and “doesn't see → doesn't know”). Second, knowledgeable agents act correctly in accordance with their knowledge (“does know → acts correctly”), while agents lacking knowledge will act incorrectly (“doesn't know → acts incorrectly”).
- Phase 3: From age 6 or later, children finally acquire a proper meta‐representational ToM and thus operate with concepts of “belief” and the like.

In each of these phases, children can thus use the strategies from the phase in question as well as from earlier ones (children in phase 2, for example, can use both perceptual access heuristics as well as reality‐based reasoning). But crucially, in a given phase, strategies from a higher phase are not available in principle. Before phase 3, from around age 6, children are thus limited in their conceptual competence in that they do not have proper meta‐representational reasoning at their disposal in principle. Based on this hypothesized sequence (reality‐based reasoning → Perceptual Access Reasoning (PAR) → belief reasoning), the PAR account predicts the following performance pattern in the TB and FB task over the course of development: Children younger than 4, confined to reality reasoning, pass TB but fail FB tasks. In contrast, 4‐ to 5‐year‐olds who can already use PAR but not yet fully fledged ToM tend to show the reverse pattern. In FB tasks, they reason based on the protagonist's lacking perceptual access when the object is relocated (“protagonist doesn't see the location change → protagonist doesn't know about it”) which leads them to answer the FB test question correctly (“protagonist acts incorrectly: searches in the wrong location”). In contrast, applying the same PAR rules in most TB tasks leads to failure. In most TB storylines, the protagonist leaves the scene and returns at some point after the object's relocation and before the test question (without anything relevant happening in the meantime) in order to hold the storyline as parallel as possible to the FB task. During her absence, the protagonist's perceptual access to the scene is interrupted and has to be established anew when she returns. On her return, she cannot see the object (“protagonist doesn't see the object → protagonist doesn't know about it”) which leads children using PAR to answer the TB test question incorrectly (“protagonist acts incorrectly: searches in the wrong location”). Only children from around 6 who are able to engage in belief reasoning proper solve both the TB and FB tasks. In this way, the PAR account, as we will call it henceforth, can elegantly account for the U‐shaped performance curve in the TB task.

Interestingly, proponents of the PAR account argue that 4‐ to 5‐year old children, even though they do not yet have meta‐representational belief concepts, do not invariably fail all TB tasks (Fabricius et al., 2010, 2021; Fabricius & Imbens‐Bailey, 2000; Fabricius & Khalil, 2003; Hedger & Fabricius, 2011). First of all, they can revert to reality‐based reasoning in certain situations that are less likely to elicit PAR and thus solve TB tasks. And they should not have any difficulty with TB tasks in which the agent does have uninterrupted perceptual access (reasoning: she sees → she knows → she acts successfully). The following performance patterns should thus be possible in children age 4−6: pass TB, fail FB (based on reality reasoning); fail TB, pass FB (based on perceptual access reasoning; involves TB tasks with interrupted perceptual access); pass both TB and FB (based on perceptual access reasoning; applies only to TB tasks in which the agent has uninterrupted perceptual access). Crucially, these are all performance patterns that may look like an expression of competent ToM reasoning. But they are not. They are merely the expression of simpler heuristics. The child at this age still lacks the competence for full‐fledged meta‐representation. And thus, some performance patterns should be impossible in principle at this age, in particular the following: children age 4−6 solve both FB and TB tasks in consistent ways where in the TB task the agent has only interrupted perceptual access. This could be in a matched pair of tasks (in story 1, protagonist A has an FB; in story 2, protagonist B has a TB with interrupted perceptual access); or it could be even more stringently in a combined task with two protagonists (protagonist A has an FB about a situation in which protagonist B has a TB with interrupted perceptual access). In contrast, such combined performance patterns in TB/FB tasks would be straightforwardly predicted by the standard picture (once relevant performance factors have been removed).

To date, many details of the PAR approach remain somewhat vague and still less than fully clear. For example, how exactly is perceptual access to be understood (Does blinking interrupt perceptual access? Does turning your head briefly?) When is perceptual access continued, and when interrupted and then newly established, and so on? Recently, proponents of the account have tried to specify some of these details and to make more specific claims regarding the factors that elicit PAR in various kinds of TB tasks (Fabricius et al., 2021). Three factors, among others, have been emphasized: First, children rely on analyzing the unfolding events and the present scenario to assess the protagonist's perceptual access. If the protagonist observes the object relocation but then leaves and returns after this relocating event, her reappearance would trigger children to perceive the scene as a new situation, initiating a new cycle of PAR regarding this second scenario (“the protagonist doesn't see the object, she doesn't know where it is, she will act incorrectly”). Second, if the protagonist's perceptual access was interrupted, this process of establishing a new cycle of PAR can be blocked by emphasizing the protagonist's past perceptual access. Highlighting past perceptual access (e.g., “remember that the protagonist saw that the object was put into location 2”) would keep children from initiating a new cycle of PAR and would make them default to their initial conclusion that the protagonist sees and knows. Conversely, TB versions without a highlighting reminder do not hinder children from initiating a new cycle of PAR and thus more likely lead to failure. Third, the standard test question in many TB tasks (“where will she look for the object *first*?”) implies that the protagonist's first search would go wrong. Proponents of the PAR account argue that this implicature encourages children to initiate a new cycle of PAR in order to end up with an answer that predicts unsuccessful behavior (Fabricius et al., 2010; see also Siegal & Beattie, 1991; Surian & Leslie, 1999).

Conceptually, it still remains unclear where the additional assumption of the account comes from, and why exactly these factors (at least the second and third) should elicit PAR, and so the suggestions may appear somewhat ad hoc and arbitrary. Relatedly, it remains unclear whether some of these factors (the third one, in particular) in real fact amount to factors of tasks pragmatics (see below). Empirically, a recent reanalysis of previous TB tasks investigated children's performance in TB tasks in light of these three hypothesized factors, and found evidence compatible with the predictions made by this modified PAR account (Fabricius et al., 2021).

In summary, the puzzling performance patterns in children older than age 4 in TB tasks raise serious questions for the standard picture of the emergence of meta‐representational ToM. According to the PAR and related conceptual competence limitation accounts, these patterns suggest that children do not have meta‐representational ToM competence before age 6 or even later.

#### 1.1.2 Performance limitation accounts: TB failure merely reflects confusing task pragmatics

An alternative view, however, is that the TB failure of children age 4−6 does not reflect competence limitations, but merely performance problems: The standard view is correct, and children this age do have full‐fledged meta‐representational ToM competence; but fail TB tasks due to pragmatic performance factors (Oktay‐Gür & Rakoczy, 2017; Rakoczy & Oktay‐Gür, 2020; Schidelko et al., 2022a; Schidelko et al., 2022b). Such accounts that emphasize conceptual competence that can get masked by pragmatic performance factors have been important and influential in many areas of cognitive science research into ToM. In developmental research with children, many studies have claimed and presented evidence that some standard ToM tasks may be artificially difficult due to pragmatic factors to do with the test questions (Helming, Strickland, & Jacob, 2014, 2016; Rubio‐Fernandez, 2013; Siegal & Beattie, 1991; Surian & Leslie, 1999; Westra, 2017). Some lifespan studies suggest that declining proficiency in some ToM tasks in older adults may reflect pragmatic performance rather than competence effects (Rakoczy, Wandt, Thomas, Nowak, & Kunzmann, 2018; Zhang, Fung, Stanley, Isaacowitz, & Ho, 2013). In comparative research with nonhuman primates, it has been forcefully argued that the many negative findings from ToM tasks with apes from the 1980s and 1990s reflect pragmatic performance limitations rather than deep competence deficits: apes do understand, to some degree, the subjective perspectives of others, but cannot express this competence in cooperative‐communicative scenarios tailed for human children; rather, their competence translates into performance best in naturalistic competitive tasks with conspecifics (Call & Tomasello, 2008). In research with neurodivergent populations, finally, it has been argued that the difficulties of autistic subjects with many standard ToM tasks may reflect pragmatic performance rather than competence limitations to do with the fact that autistic subjects are asked to take the perspective of dissimilar (neurotypical) rather than similar (other autistic) subjects (Cheang, Skjevling, Blakemore, Kumari, & Puzzo, 2024).

The present performance limitation accounts is a member of this big family of pragmatic performance limitation accounts across the cognitive sciences of ToM (and beyond). In the present context, the specific pragmatic task analyses suggest that TB tasks may be particularly challenging for children between the ages of 4 and 10 because they pose strange and pragmatically confusing test questions. In particular, it has been suggested that the combination of various factors makes TB test questions pragmatically odd, infelicitous, and irritating. First, the TB question is an academic test question—quite different from regular questions. Regular questions are asked when the speaker lacks information and seeks it from the interlocutor. However, academic test questions (as the TB question) have a more complex structure. They aim to assess if the interlocutor knows something the speaker knows herself. This special question format may be difficult for young children to understand (e.g., Siegal, 1999). Second, the TB tasks follow a very trivial storyline: the child, the experimenter, and the story protagonist witness that an object is moved from one to another location. The answer to the question where the protagonist will go to get the object is thus obvious and common knowledge, making it challenging to comprehend why someone would ask and test what is so obvious and mutually known. Third, this might be particularly pronounced in the context of TB tasks as the TB test questions pertain to a subjective belief or a belief‐based action. Typically, questions regarding action prediction, explanation, or belief ascription have a clear pragmatic point: they are posed only when there is a potential for error and misrepresentation. Consequently, the test questions “What does she believe?” or “Where will she look for her object?” suggest the possibility of an alternative perspective or misrepresentation. However, the storyline within the TB task lacks evident opportunities for error or misrepresentation. As a result, children may feel as if they have missed some information and seek out possible alternative perspectives within the scenario in order to make sense of the question. Therefore, the combination of these factors in TB tasks can create pragmatic confusion in children with nascent pragmatic sensitivity. Children before age 4, before they have acquired basic meta‐representational capacities and thus the foundations of complex pragmatic sensitivity, do not get confused by such questions since they simply read and answer them literally. From age 4 on, however, once they do engage in more complex pragmatic considerations, children get pragmatically confused. Only later, from age 8−10, when children have acquired yet more complex meta‐representational and pragmatic capacities that allow them to make sense of any arbitrarily complicated speech act, do children overcome the pragmatic confusions and answer the TB questions correctly in spontaneous unaided ways.

The first evidence for this pragmatics account comes from recent studies that manipulated pragmatic task factors in children aged 4−10. First of all, children showed no difficulties with the TB task when presented with a fully nonverbal variant of the task that eliminated any (academic and trivial) test question (Rakoczy & Oktay‐Gbr, 2020, Exp. 1). Second, children had no difficulty with verbal TB questions when their triviality was explicitly highlighted (“I'll ask you a baby question”) (Rakoczy & Oktay‐Gür, 2020, Exp. 5). Third, children performed much better, indeed very competently, once the TB tasks were made more complex and thereby more natural. One agent with a TB was contrasted, in the same scenario, with another agent who had an FB (Oktay‐Gür & Rakoczy, 2017, Exp. 3). Asking about the agent's TB now had a point since the possibility of misrepresentation was given by the second (FB) agent. Converging evidence for the importance of the third factor (reference to subjective perspective of a rational agent) comes from a study in which children showed no difficulties in a parallel task that involves a trivial, academic test question that pertains to a nonmental perspective (Schidelko et al., 2022a).

Taken together, these results suggest that the combination of certain task features makes TB pragmatically odd and, therefore, demanding for children from age 4 on. Once these factors are removed or weakened, children can bring their meta‐representational ToM competence to bear in both TB and FB performance.

In summary, children's puzzling failure in TB tasks prima facie seems to challenge the standard picture of the emergence of meta‐representational ToM around age 4. Two different kinds of approaches aim at explaining this puzzling performance pattern. Conceptual competence limitation accounts claim that this pattern indeed is incompatible with the standard picture: it reveals that children do not yet operate with meta‐representation until much later but use simpler heuristics. In contrast, performance limitation accounts claim that the puzzling TB failures merely reflect difficulties to do with task pragmatics. The standard picture is correct and 4‐year‐olds do have meta‐representational ToM, but they fail to exhibit it in some pragmatically demanding tasks. Both types of accounts have investigated developmental trajectories in TB performance from their respective perspectives and have yielded evidence compatible with their predictions. What is missing so far is a study that tests these accounts against each other in direct and systematic ways.

## 2 Rationale of the present study

The present study is the first attempt at implementing such a systematic investigation with a direct contrast. To this end, in a 2×2 design, we devised TB tasks in which we independently varied factors deemed to be crucial by the two types of account. First, those features of the TB task that should be crucial in eliciting perceptual‐access‐heuristic‐mistakes according to the PAR account were varied (see the three features mentioned above: protagonist movement, highlighting, first‐look test question). In PAR+ conditions—those that according to the account should elicit most TB mistakes based on perceptual access heuristics, the protagonist left and returned, there was no highlighting of the fact that she had witnessed the relocation, and the “look first” question was asked. Conversely, in PAR− conditions, the protagonist stayed, there was highlighting, and a more neutral “where will she look”‐question was asked (without “first”).

Second, the factors deemed to be crucial by the pragmatic performance limitation account were varied in the following ways: in the Trivial+ conditions, a normal TB task was administered that posed a trivial, academic test question about the subjective perspective of an agent. In the Trivial− conditions, triviality was reduced by making the task more complex and the question about the subjective perspective of the main agent more natural: this agent (with a TB) was accompanied by another protagonists who failed to witness the crucial relocation of the object and thus had an FB. The test questions now referred to both agents (TB and FB, respectively).

### 2.1 Competing predictions

Crucially, the predictions about children's performance in these different versions, and more generally, of the stability and potential malleability of these patterns differ significantly between the accounts. Competence deficit accounts in general, and the PAR account specifically, argue that the patterns stem from fundamental limitations in conceptual ToM competence. This implies, first, performance pattern should remain consistent even when irrelevant task features change. Second, this implies a consistent negative correlation of FB and TB performance (for those TB tasks with interrupted perceptual access of the agent that elicit PAR) until age 6 or older: children either engage in reality‐based reasoning, solving TB but failing FB tasks; or they engage in perceptual access reasoning, solving FB but failing TB tasks. But children before age 6 should be incapable in principle—due to their conceptual competence limitations—of solving pairs of FB and TB questions within a given scenario (the only exception should be those tasks where the TB condition does not involve an interruption of the perceptual access of the agent. Here, children engaging in PAR may be able to solve both TB and FB conditions). In contrast, pragmatic performance limitation accounts make quite different predictions: the perplexing performance patterns (children from age 4, once they pass FB tasks, begin to fail TB tasks, with negative correlations between TB and FB) reflect merely performance limitations in TB tasks due to pragmatic task factors. Therefore, children will reveal proficient performance in both FB and TB tasks once these performance factors have been removed or reduced. In particular, children should be able to perform competently in pragmatically modified combined FB and TB tasks in all kinds of conditions—including those in which the PAR account would predict principled TB failure (involving interrupted perceptual access of the agent). The clearest case of competing predictions thus pertains to the crucial conditions in which PAR‐eliciting factors are present (agent leaves, no highlighting, look first question) but the pragmatic performance factors are reduced: for this condition, the PAR account predicts in principle incompetence, whereas the pragmatic performance factor account predicts success.

## 3 Methods

The study including details on the design, sample size, and analysis is preregistered on *AsPredicted* (https://aspredicted.org/9cs3‐jjv9.pdf). It was conducted in accordance with the 1964 Helsinki declaration, its later amendments, and comparable ethical standards. It involved no invasive or otherwise ethically problematic techniques and no deception (and, therefore, according to National jurisdiction, did not require a separate vote by a local Institutional Review Board; see the regulations on freedom of research in the German Constitution (§ 5 (3)), and the German University Law (§ 22)).

### 3.1 Participants

The final sample included 165 children^2^ between 4 and 7 years (48−95 months). Children were equally distributed across age groups and conditions (for details, see Supplement). Eight additional children were tested but excluded from the analysis because of technical issues during the test session (*n* = 1), missing video recording (*n* = 2), abortion of the test session (*n* = 1), uncooperative behavior (*n* = 3), and recent participation in a similar study (*n* = 1).

Parents/legal guardians gave verbal consent for participation before the testing was started. Verbal consent was recorded and stored separately from the recording of the test session.

### 3.2 Design

**Fig. 1:**
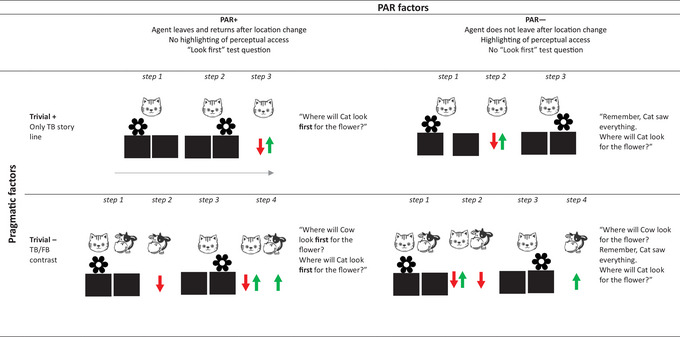
Schematic structure of the change‐of‐location true belief task for all conditions: the cat first sees the flower being put in Box 1 and later observes the flower being removed to Box 2. In the “PAR+” conditions (left column), the Cat first sees the object being moved and then leaves the scene, the Cat's perceptual access is not highlighted and the test question does ask for “first‐look.” In the “PAR−” conditions (right column), the Cat first leaves the scene and then sees the object being moved after her return, the Cat's perceptual access is highlighted and the test question does not ask for “first‐look.” In the “Trivial−” conditions (lower row), the TB test question is contrasted with an FB test question (about the Cow), while the “Trivial+” conditions (upper row) do not involve this contrast.

The design followed a 2 (Pragmatic factors: Trivial+ or Trivial−) × 2 (perceptual access reasoning factors: PAR+ or PAR−) between‐subject design. TB versions that were supposed to elicit PAR involve the three features introduced above (1. the protagonist leaves and returns after the location change, 2. no highlighting of the protagonist's past perceptual access, and 3. “look first” test question). TB versions which are supposed to less likely elicit PAR do not involve these features (1. the protagonist does not leave after the location change, 2. the protagonist's past perceptual access is highlighted, and 3. test question does not ask for *first* look: “Where will protagonist look for object?”). TB versions with reduced pragmatic performance limitation factors reduced the triviality of the TB test question by contrasting the TB test question with a test question about a second protagonist who holds an FB. TB versions that do not have reduced pragmatic performance limitation factors do not involve this contrast. This results in four TB versions depicted in Fig. 1. Each child received two TB trials of the same version (with varying characters and objects) and one FB trial.

### 3.3 Procedure

The tasks were presented remotely (on a computer or tablet screen) in an interactive online study via the video conferencing platform BigBlueButton (for details on the remote test setting, see Schidelko, Schünemann, Rakoczy, & Proft, 2021). One of three female experimenters (E) presented the tasks by screen‐sharing short video clips (created with the video animation software VyondTM © GoAnimate, Inc., 2021). The child watched the video clips while E read out the storylines (for detailed information on the script, see Supplement). At the end of each storyline, E asked the control and test questions. Children first received one trial of the classic FB task and after that, two TB trials of the same version.

#### 3.3.1 FB task

In the standard change‐of‐location task (Wimmer & Perner, 1983), the boy puts his candy in Box 1 and leaves before the girl enters and moves the candy to Box 2. The girl leaves again and the boy re‐enters the scene before E asks the test question (“Where will the boy look (first) for the candy?”). The exact wording of the test question in the FB task corresponds to the test question from the respective TB condition. In the “PAR+” conditions (conditions 1 and 3), the FB test question does ask—as the TB test question—for “first‐look.” In the “PAR−” conditions (conditions 2 and 4), the test question does not ask for “first‐look.”

#### 3.3.2 TB task

In all four conditions of the TB task, the cat first sees the flower being put in Box 1 and later observes the flower being removed to Box 2. In the PAR+ conditions (conditions 1 and 3), the Cat first sees the object being moved and then leaves the scene, the Cat's perceptual access is not highlighted and the test question does ask for “first‐look.” In the PAR− conditions (conditions 2 and 4), the Cat first leaves the scene and then sees the object being moved after her return, the Cat's perceptual access is highlighted and the test question does not ask for “first‐look.” In the Trivial− conditions (conditions 3 and 4), the TB test question is contrasted with an FB test question (about the Cow),^3^ while the Trivial+ conditions (conditions 1 and 2) do not involve this contrast.

## 4 Results

### 4.1 Descriptive results

**Fig. 2:**
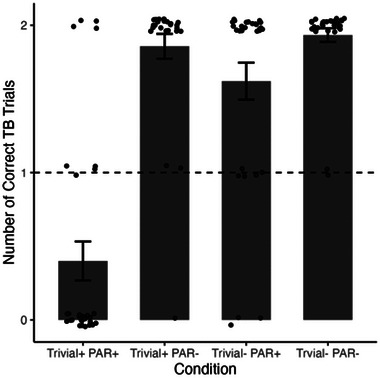
Mean number of TB trials that were answered correctly as a function of condition for FB passers (*n* = 117). Points depict the performance of individual participants. Dashed line depicts the chance level.

For the main analysis, only children who passed the standard FB task in the beginning of the test session were included (*n* = 117) as these children are expected to fail the TB task (Fabricius et al., 2010, 2021; Oktay‐Gür & Rakoczy, 2017). In this subsample, children passed 72.22% of the TB trials. The performance in the TB task, however, varied across conditions (see Fig. 2). Children performed significantly below chance level in the first condition (Trivial+/PAR+: *M* = 0.4, *V* = 54, *p* < .001) and significantly above chance level in the three other conditions (*V*s > 262, *p*s < .001).

### 4.2 Main analysis

To analyze the impact of the pragmatic triviality factor and PAR factors on the probability of success in TB trials, we fitted a Generalized Linear Mixed Model with a binomial error structure. As dependent variable, children's answer (“correct” or “incorrect”) on each TB trial was used. Pragmatic task triviality factors (“Trivial+” or “Trivial−”) and PAR factors (“PAR+” or “PAR−”) were included as main predictors in the full model.^4^ Additionally, children's age in months and the trial number (“TB1” or “TB2”)^5^ were included as control predictors. To account for repeated measures, children's ID was added as random effect.

Overall, the main predictors had a clear impact on the success in the TB task. The model comparison of the full model and a reduced model lacking the main predictors was significant (*χ* ^2^ = 56.31, *p* < .001). More specifically, children were more likely to answer the TB question correctly when the pragmatic triviality was reduced and PAR was not elicited. In contrast, neither the children's age nor the trial number had a significant effect. The model results are depicted in Table 1.

**Table 1:** Model results on children's success in TB trials

	Estimate	SE	95% CI		*z*	*p*
Intercept	−9.22	4.50	−18.58	−6.30	−2.12	.03
Age in months	0.03	0.06	−0.04	0.06	0.53	.59
Trial number	−0.31	0.79	−13.62	3.25	−0.39	.70
PAR factors (PAR±)	16.13	2.19	17.24	40.49	7.37	<.001
Pragmatic triviality (Trivial±)	15.06	1.97	16.93	40.62	7.63	<.001

*Note*. Estimates, standard error (SE), *z*‐values, and *p*‐values of binomial mixed effects model on children's success in TB trials with age (in months), trial number, PAR factors, pragmatic triviality factors, and random intercepts for participants. *N*_observations_ = 234 trials. The 95% confidence intervals (CI) were obtained via bootstrapping with 1000 boots.

### 4.3 Secondary analysis

**Fig. 3:**
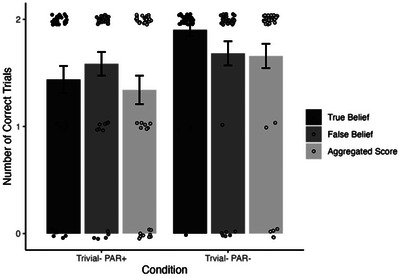
Mean number of trials in which TB and FB questions were answered correctly and aggregate scores (of both TB and FB within one trial) as a function of condition (note that the chance level of guessing correctly differed between TB/FB [chance level = 50%, i.e., 1] and the aggregate score combining both measure [chance level = 25%, i.e., 0.5]).

In a secondary analysis, we analyzed the performance of participants who were tested in one of the two Trivial− conditions that involved both a TB and an FB within the same storyline. This analysis is pivotal since for these conditions pragmatic performance limitation accounts and PAR accounts make clearly competing predictions. According to pragmatic performance limitation accounts, children should be able to perform competently in pragmatically modified combined FB and TB tasks in all kinds of conditions—including those in which the PAR account would predict TB failure (involving interrupted perceptual access of the agent). For this part of the analysis which focused on the relation of FB and TB performance, we also included children who failed the separate FB task presented at the beginning of the test session.^6^ Children's performance in response to the TB and FB questions (as well as their combined performance in response to both types of questions per trial) as a function of condition are depicted in Fig. 3.

First, the analysis showed that children performed significantly above chance level in the FB test question in both conditions (Trivial−/PAR+: *M* = 1.59, Trivial−/PAR−: *M* = 1.68, *p*s < .001).

Second, we analyzed the relation of FB and TB task performance within the same task (see Table 2). Correlational analyses between TB and FB performance within the same task controlled for children's age yielded the following results. Across the two Trivial− conditions, TB and FB performance was positively correlated (Trial 1: *r* = .54, *p* < .001; Trial 2: *r* = .33, *p* < .001). When computed separately for conditions, children's performance correlated significantly in the Trivial−/PAR+ condition (Trial 1: *r* = .62, *p* < .001; Trial 2: *r* = .44, *p* < .001) and in the first trial of the Trivial−/PAR− condition (Trial 1: *r* = .46, *p* < .001; Trial 2: *r* = .22, *p* = .16).^7^

**Table 2:** Contingency between performance in FB and TB test question when tested within one task (Trivial− conditions) including all children (i.e., children who passed and failed the separate FB task, *n* = 165)

		TB trial 1		TB trial 2	
		Incorrect	Correct	Incorrect	Correct
**FB** (in the corresponding task trial)	Incorrect	2	5	5	2
	Correct	0	34	6	28

Third, we computed aggregate scores that took into account whether children solved both TB and FB within the same trial. A trial only received an aggregate score “correct” if children answered both TB and FB in this trial correctly (with a chance level of guessing correctly of 1/4). Chance level comparisons showed that aggregated score levels (summed over two trials) were significantly above chance (0.5) in both conditions (*V*s>770, *p*s < .001).

## 5 General discussion

The present study investigated an interesting and potentially dramatic test case for rich versus lean theories of early ToM development. This test case is the puzzling finding from many previous studies that children from the age of 4, once they master FB tasks, begin to fail TB control tasks. Do these mistakes in TB tasks speak for lean theories of ToM development? Do they indicate that children use simple (perceptual access) heuristics and actually lack the conceptual competence (meta‐representational ToM) standardly ascribed to them on the basis of their performance in FB tasks? Or are these findings compatible with rich theories of ToM development? Do the mistakes children make merely indicate pragmatic performance factors that mask children's meta‐representational competence? The present study was the first to systematically test the lean competence limitation and the rich performance limitation accounts against each other. To this end, 4‐ to 7‐year‐olds were tested on different versions of the TB task that systematically varied in task features deemed relevant by the two accounts.

### 5.1 Summary of the main results

The main results were the following: First, in a kind of baseline condition in which neither perceptual access nor pragmatic factors were specifically modified, the standard TB failure in children from age 4 on could be replicated (children who were at ceiling in FB performance performed at levels below chance on TB tasks, with only 20% success rate). Second, once perceptual access factors (deemed crucial by the competence limitation account) and/or pragmatic task factors (deemed relevant by the performance limitation account) were modified, the TB failures disappeared, and children performed at above chance levels. Both factors independently predicted success. Third, in those conditions that involved two protagonists (one with a TB, the other with an FB) and thus combined FB/TB question pairs, children competently answered both types of questions correctly at levels above chance (correctly ascribing an FB to one, and a TB to the other agent in the same scene). Fourth, this was also the case, importantly, in conditions in which the perceptual access competence limitation account predicted that it should not be the case in principle (the condition with reduced pragmatic task factors, but interrupted perceptual access of the agent to finally hold a TB).

### 5.2 Implications for the debate between competence versus performance limitation accounts

What are the implications of these results for the debate between competence and performance limitation accounts? Overall, many of the present findings are compatible with both types of accounts. But crucially, some findings are incompatible with the principles of the PAR competence limitation account under investigation; they thus speak in favor of the performance limitation account. As a background, it is here important to note, first of all, that the two types of accounts are not symmetrical in their structure and their predictions. The rich pragmatic performance limitation account claims that children from age 4 do have meta‐representational ToM competence in principle; but in practice, pragmatic (and potentially other) performance factors may mask this competence in many TB tasks (Oktay‐Gür & Rakoczy, 2017; Rakoczy & Oktay‐Gür, 2020; Schidelko et al., 2022a; Schidelko et al., 2022b). Crucial performance factors pertain to confusing task pragmatics: TB tasks involve trivial, academic test questions about agents’ subjective perspective in situations in which such discourse seems to lack its typical point (there is not even the possibility of misrepresentation, error, etc. which usually provide the background for asking about subjective perspectives in contrast to objective reality). Once these performance factors are modified (removed or reduced), children age 4 and beyond should not have any particular problems with TB tasks, now performing equally proficiently in FB and TB tasks. Importantly, such performance limitation accounts are not necessarily exhaustive regarding relevant performance factors: The claim is that children have a competence that may get masked. Once pragmatic performance factors are removed, children perform competently. The claim is not, however, that they *only* perform competently when these performance factors are removed. Reducing the pragmatic performance factors described here is sufficient for making the TB failures disappear, but not necessarily. There may well be others that are relevant as well. In other words, the fact that children perform well when pragmatic performance factors are removed, and also once other factors (perceptual access, in the present case) are modified, does not mean that the pragmatic performance limitation account is wrong (rather, at most, that it is incomplete).

On the other side, the lean competence limitation account claims that children before age 6 do not, in principle, have full‐blown meta‐representational competence (Fabricius et al., 2010, 2021). Rather, they merely have simpler heuristics in use that mimic, under certain circumstances, meta‐representational ToM: children between age 4 and 7, roughly, use perceptual access heuristics of the sort: if an agent has (had) perceptual access to relevant events in a scene, she will act successfully; if she has had no, or interrupted perceptual access, she will act unsuccessfully. Manipulating the circumstances affects performance. In particular, modifying TB tasks in such ways that perceptual access heuristics happen to produce the right kinds of results in both FB and TB tasks (the agent always has interrupted perceptual access only, etc.) will boost performance. But there are clear limits to which types of tasks children this age can master given their lack of meta‐representational competence. One crucial limit, for example, pertains to pairs of FB and TB tasks in which two agents both have interrupted perceptual access but where one ends up with an FB and the other with a TB (the PAR+/Trivial− condition in the present study). With PAR, it should be impossible in principle to solve such pairs of FB/TB questions. Empirical findings that such limits, predicted by the PAR account in principle and on theoretical grounds, do not hold would thus provide counterevidence against the account.

Against this background, what do the present findings show? Clearly, one part of the findings is compatible with both types of accounts. That the basic TB failure pattern could be reproduced in a baseline condition (without specific modifications of perceptual access or pragmatic factors) and that this failure could be made to disappear under some modifications is in line with both accounts. More specifically, that modifying perceptual access factors makes a difference speaks for the PAR account; that modifying pragmatic factors makes a difference speaks for the pragmatic performance limitation account. That the modification of perceptual access factors made a difference does not directly speak for the pragmatic performance limitation account—but, crucially, it also does not speak against it. This account claims that children have meta‐representational competence that may get masked by performance factors, pragmatic or otherwise.

In contrast, one set of findings clearly *does* speak against the PAR account: that the modification of pragmatic task factors alone, in the PAR+/Trivial− condition in particular, made a difference is a finding that is incompatible with the logic of the PAR account. This account claims that children age 4−6 or so lack meta‐representational competence and only operate with simpler heuristics. These may mimic true meta‐representational performance in some circumstances, but this has clear limits. What should be impossible with such heuristics is solving combined FB/TB tasks in which the relevant perceptual access features have not been modified (the agent still has interrupted perceptual access, etc.). But these are exactly the cases (in the relevant PAR+/Trivial− condition) that children in the present study were very proficient at.

### 5.3 Conclusions and outlook

Overall, the present results thus allow us to make progress regarding the basic question addressed here: What is the cognitive foundation of the bizarre patterns of failure in TB tasks in children from age 4? Does it reflect a deep (meta‐representational) competence limitation, or merely a more superficial performance limitation? The present findings clearly speak for the latter. They are compatible with (partly speak directly for, and partly are consistent with) the pragmatic performance limitation account. In contrast, the present findings as a whole do not fully align with the PAR account understood as a competence limitation account. In a broader context, these findings speak for the importance of taking into account pragmatic performance factors when interpreting the results from ToM or other social‐cognitive tasks with various populations (see Helming et al., 2014; Rubio‐Fernandez, 2013; Westra, 2017)

At the same time, the present findings leave open a number of interesting possibilities and raise a number of interesting questions to be addressed in future research: First of all, the present findings are perfectly compatible with some version of a perceptual access account understood as a performance limitation account: children from age 4 have meta‐representational competence, but may revert to simpler heuristics under certain circumstances to do with the agents’ perceptual access. One version of such an account may take inspiration from recent “knowledge first” or factive ToM accounts (e.g., Nagel, 2017; Phillips et al., 2021). Children may initially be able to ascribe only factive mental states such as perception and knowledge, and this primary and primordial form of factive ToM remains in operation throughout the lifespan as the basic and default mode of operation. Only later (from age 4) do children then come to acquire full meta‐representational competence to ascribe nonfactive attitudes like belief. Some cases to do with perceptual access may be confusing for thinkers who have both factive and nonfactive ToM since they involve conflicting intuitions. These are cases where an agent's perceptual access to relevant facts is interrupted in such ways that her beliefs remain true, while we would be hesitant to ascribe knowledge to her (imagine, for example, that the agent sees an object at location A, then leaves the room, and in her absence, the object is moved from A to B to C, etc., and then much later back to A again. These are very similar to so‐called “Gettier” cases in epistemology; Gettier, 1963). Such cases may thus be difficult not because children do not have the competence to ascribe beliefs, but because their capacity for belief ascription (“She truly believes…”) and for knowledge ascription (“but she still does not know…”) yield conflicting intuitions.

Second, what is the nature of the various factors highlighted by the PAR account? May some of these factors actually turn out to be pragmatic task demands in the end? For one of these factors highlighted by Fabricius et al. (2021), this is indeed quite obvious: Asking the “Where will she look first?”‐question has long been analyzed in Gricean ways as conveying implicatures such that the first search may not be successful and a second search may thus be needed (Siegal & Beattie, 1991; Surian & Leslie, 1999). Originally, this point has been made in debates about potential false negatives in standard FB tasks: Here, asking “where will she look?” may be misunderstood by young children in normative terms as “where ought she look?” or “where will she find…?” Based on such misunderstandings, children may answer the test question incorrectly by claiming the agent will look for the object where it really is—meaning “she will search successfully” or “she ought to search” at this location. The “look first” modification may then be seen as a way to remove these pragmatic task demands (the empirical situation regarding the question whether the “first” indeed reduces task demands is mixed and complicated; see Wellman et al., 2001). Conversely, in the TB task, asking the “look first?” question may carry pragmatically confusing implicatures. It suggests the plausibility of a second search and thus the plausibility of misrepresentation (why would someone ask this “look first?” question otherwise?). In order to make sense of the question and its implicature, children may then assume they must have missed something and infer that it cannot be as simple as it appears (the agent has full informational access and will thus search exactly once, and successfully…). Relatedly, it is possible that another factor listed by the PAR account and used here—highlighting the premise about the agent's complete and veridical informational access—may be closely intertwined with such pragmatic processes: If children are pragmatically confused and try to make sense of an irritating question, they may come to think that they must have missed something, such that the agent missed some crucial event after all and ends up with an FB (“why else would the experimenter ask me such questions?”). Such reinterpretations become all the less plausible and possible, however, the more the relevant premises are made explicit and highlighted. Clearly, at the current stage, these are merely speculative thoughts about the possible underlying pragmatic nature of (some of) the task factors deemed relevant by the PAR account. Future research will need to transform these speculations into stringent examinations.

A third and final question for future research is the following: If, as the current findings suggest, we can best explain why children from age 4 begin to fail TB tasks in terms of pragmatic performance limitations, then how can we explain the other end of the U‐curve? Why do children from age 8−10 then cease to have any trouble with any type of TB task, however, trivial and pragmatically odd? Clearly, we currently do not know. The general line of explanation in pragmatic terms would have to go roughly like this: once children acquire basic meta‐representational competence and thus pragmatic sensitivity from age 4, this opens up new ways of being confused by infelicitous discourse, irritating implicatures, and so on. Some levels up, however, with ever more complex recursive meta‐cognition and thus higher‐order pragmatic sensitivity, and possibly aided by ample experience with strange discourse in educational settings (academic test questions in school…), children finally come to understand that people can engage in all kinds of confusing discourse for various reasons (they may ask themselves and others, for example, such silly questions “Is this my hand?” and “How do I know this is my hand?” in epistemology classes, etc.). We would thus expect that the development of higher‐order meta‐representation (Schidelko et al., 2022b; Wilson et al., 2022) and of advanced pragmatic skills in other areas such as understanding complex indirect speech acts (e.g., Happé, 1994) go hand in hand with children overcoming the pragmatic performance limitations in the TB tasks. Systematic and stringent future research will need to carefully delineate such developmental trajectories. In a broader context, this research may also be helpful for understanding how pragmatic performance factors work and mask competence in other areas of social cognitive science.

## Funding

This work was supported by the Deutsche Forschungsgemeinschaft (DFG, German Research Foundation); SFB 1528: Cognition of Interaction.

## Supporting information







## Notes

1 The standard picture has come under pressure from various directions: “Boosters” have argued that it underestimates competence, and that even infants have some (possibly implicit) ToM (Baillargeon, Scott, & He, 2010). Here, we focus on the opposite (“scoffer”) skepticism: that the standard picture overestimates competence (for a more thorough treatment of booster and scoffer concerns and their relations, see Rakoczy, 2017).

2 A sample size of 160 children was preregistered but due to a scheduling error, five more children participated in the study.

3 For this contrast, the FB test question about the cow was always asked first. A previous study counterbalanced the order of the contrasting questions (question about protagonist holding a TB or a FB first) leading to good performance in the TB test question irrespective of the order (Oktay‐Gür & Rakoczy, 2017). Therefore, we kept the order of the contrasting questions constant here for simplicity's sake.

4 Pragmatic task triviality factors (“Trivial+” or “Trivial−”) and PAR factors (“PAR+” or “PAR−”) were included as main predictors without an interaction term of the two variables as there was no theoretically driven a priori reason to assume an interaction. Including the interaction term does not change the results (see Supplement).

5 The control variable *Trial Number* was not included as predictor in the preregistered analysis. The model results do not differ from the preregistered model without this control variable.

6 For correlation analysis on FB and TB performance tested in separate tasks, see Supplement.

7 Lacking correlation effects probably result from ceiling effects in TB performance.
