# Supplementary material for: Why Do Children From Age 4 Fail True Belief Tasks? A Decision Experiment Testing Competence Versus Performance Limitation Accounts
Source: Cogn Sci. 2025 Jun 6;49(6):e70069. doi: 10.1111/cogs.70069 (PMC12143423; doi:10.1111/cogs.70069)
Supplement: Supplementary file 1 [file COGS-49-e70069-s001.docx]

**Supplement**

**Methods**

*Participants*

Distribution across age groups

| Group | Number | Mean age in months (and SD) | Number female | Number male | Number non-binary/not specified |
| --- | --- | --- | --- | --- | --- |
| Four-year-olds  (48-59 months) | 43 | 52.74 (3.70) | 20 | 23 | 0 |
| Five-year-olds  (60-71 months) | 40 | 66.13 (3.53) | 20 | 20 | 0 |
| Six-year-olds  (71-83 months) | 41 | 78.00 (3.54) | 20 | 21 | 0 |
| Seven-year-olds  (84-95 months) | 41 | 88.88 (2.45) | 21 | 20 | 0 |

Distribution across conditions

| Group | Number | Mean age in months (and SD) | Number female | Number male | Number non-binary/not specified |
| --- | --- | --- | --- | --- | --- |
| Trivial+/PAR+ | 41 | 71.39  (14.57) | 24 | 16 | 1 |
| Trivial+/PAR- | 42 | 70.60 (14.08) | 11 | 31 | 0 |
| Trivial-/PAR+ | 41 | 71.36  (14.19) | 24 | 17 | 0 |
| Trivial-/PAR- | 41 | 71.63  (13.88) | 21 | 20 | 0 |

*Procedure*

Full Script

|  | Trivial+/PAR+ | Trivial+/PAR- | Trivial-/PAR+ | Trivial-/PAR- |
| --- | --- | --- | --- | --- |
| Intro | E: Look, there is the cat! Hi Cat!  Cat: Hello!  E: Look, Cat and (name of the child), what else is here! A green box… and a purple box.  E: Look, Cat and (name of the child), what else is here! A flower.  Cat: Oh great, a flower!  E: Alright, we put the flower into the purple box.  Cat: ok! | | E: Look, there is the cat! Hi Cat!  Cat: Hello!  E: And look, there is the cow. Hi Cow!  Cow: Hello!  E: Look, Cat, Cow and (name of the child), what else is here! A green box… and a purple box.  E: Look, Cat, Cow and (name of the child), what else is here! A flower.  Cat: Oh great, a flower!  Cow: Oh nice, a flower!  E: Alright, we put the flower into the purple box.  Cat: ok!  Cow: ok! | |
| Protagonist movement before location change | *Cat stays* | *Cat leaves*  *Cat:* “I have to leave now. See you!” | *Cat stays*  *Cow leaves*  Cow: “I have to leave now. See you!” | *Cat and Cow leave*  Cat: “I have to leave now. See you!”  Cow: “I have to leave, too. See you!” |
| Pause |  | E: Okay, (name of the child), cat is gone now. Let’s wait a minute. | E: Okay, (name of the child), cow is gone now. Let’s wait a minute. | E: Okay, (name of the child), cat and cow are gone now. Let’s wait a minute. |
| Protagonist return before location change |  | *Cat returns:* “Hi, I’m back!”  E: “Hi Cat!” |  | *Cat returns*  *Cat:* “Hi, I’m back!”  E: Hi Cat! |
| Location change | E: Oh, see Cat and (name of the child), what’s happening now. We take the flower out of the purple box and put it into the green box.  Cat: Ah, okay. | | | |
| Protagonist movement after location change | *Cat:* “I have to leave now. See you!”  *Cat leaves* | *Cat stays* | *Cat:* “I have to leave now. See you!”  *Cat leaves* | *Cat stays* |
| Protagonist return after location change | *Cat returns*  Cat: “Hi, I’m back!”  E: “Hi Cat!” |  | *Cat and Cow return*  Cat: “Hi, I’m back!”  Cow: “Hi, I’m back!”  *E: Hi, Cat and Cow!* | Cow returns  Cow: “Hi, I’m back!”  E: “Hi Cow!” |
| Control questions | Control question 1: “Where did I put the flower in the beginning?”  Control question 2: “Where is the flower now?”  *(if wrong: “think about it again” and repeat question)* | | | |
| FB questions | *No FB question* | *No FB question* | FB question: Where will Cow look **first** for the flower? | FB question: Where will Cow look for the flower? |
| Highlight | *No highlight* | E: Remember, Cat saw everything. | *No highlight* | E: Remember, Cat saw everything. |
| TB test question | TB question: Where will Cat look **first** for the flower? | TB question: Where will Cat look for the flower? | TB question: Where will Cat look **first** for the flower? | TB question: Where will Cat look for the flower? |

**Analysis**

*Additional analysis on the relation of TB and FB performance tested in two separate tasks*

Across all conditions, children’s performance in the FB task (tested at the beginning of the test session) and the TB tasks did not correlate significantly when controlled for children’s age in months (*r* = -.03, *p* = .74). However, when analyzed separately for the four conditions, TB and FB performance correlated in the Trivial-/PAR+ condition (*r* = .27, *p* = .09) but not in any other condition (Trivial+/PAR+: *r* = -.27, *p* = .1, Trivial+/PAR-: *r* = -.13, *p* = .43, Trivial-/PAR-:  *r* = .1, *p* = .56). For the two latter, however, these results are difficult to interpret as children performed close to ceiling in the TB tasks.

*Additional Model Analysis with Interaction Term*

In the original model analysis, pragmatic task triviality factors (“Trivial+” or “Trivial-“) and PAR factors (“PAR+” or “PAR-“) were included as main predictors without an interaction term of the two variables as there was no theoretically-driven a priori reason to assume an interaction. However, in an additional analysis, we fitted the same model with the added interaction term of pragmatic task triviality factors (“Trivial+” or “Trivial-“) and PAR factors (“PAR+” or “PAR-“) leading to the same results.

Model Results

|  | Estimate | SE | z | p |
| --- | --- | --- | --- | --- |
| Intercept | -9.54 | 4.66 | -2.05 | .04 |
| Age in months | 0.04 | 0.06 | 0.70 | .48 |
| Trial Number | -0.30 | 0.78 | -0.39 | .70 |
| PAR factors (PAR+/-) | 15.75 | 3.05 | 5.16 | <.001 |
| Pragmatic triviality (Trivial+/-) | 14.35 | 2.83 | 5.07 | <.001 |
| Interaction PAR*Prag | -13.11 | 4.31 | -3.04 | .002 |

*Note.* Estimates, standard error (SE), *z*-values, and *p*-values of binomial mixed effects model on children’s success in TB trials with age (in months), trial number, PAR factors, pragmatic triviality factors, the interaction of PAR and pragmatic triviality factors and random intercepts for participants. *N*_observations_= 234 trials.
